# Reconstructing Phylogeny by Aligning Multiple Metabolic Pathways Using Functional Module Mapping

**DOI:** 10.3390/molecules23020486

**Published:** 2018-02-23

**Authors:** Yiran Huang, Cheng Zhong, Hai Xiang Lin, Jianyi Wang, Yuzhong Peng

**Affiliations:** 1 School of Computer and Electronics and Information, Guangxi Universities Key Laboratory of Parallel and Distributed Computing, Guangxi University, Nanning 530004, China; 2 School of Computer Science and Engineering, South China University of Technology, Guangzhou 510006, China; 3 Guangxi Colleges and Universities Key Laboratory of Data Science, Guangxi Teachers Education University, Nanning 530001, China; jedison@163.com; 4 Guangdong Key Laboratory of Popular High Performance Computers, Shenzhen Key Laboratory of Service Computing and Applications, Shenzhen 518060, China; 5 Faculty of Electrical Engineering, Mathematics and Computer Science, Delft University of Technology, Mekelweg 4, 2628 CD Delft, The Netherlands; h.x.lin@tudelft.nl; 6 School of Chemistry and Chemical Engineering, Guangxi University, Nanning 530004, China; jianyiwang@gxu.edu.cn

**Keywords:** metabolic pathway alignment, functional module mapping, phylogenetic tree, union graph

## Abstract

Comparison of metabolic pathways provides a systematic way for understanding the evolutionary and phylogenetic relationships in systems biology. Although a number of phylogenetic methods have been developed, few efforts have been made to provide a unified phylogenetic framework that sufficiently reflects the metabolic features of organisms. In this paper, we propose a phylogenetic framework that characterizes the metabolic features of organisms by aligning multiple metabolic pathways using functional module mapping. Our method transforms the alignment of multiple metabolic pathways into constructing the union graph of pathways, builds mappings between functional modules of pathways in the union graph, and infers phylogenetic relationships among organisms based on module mappings. Experimental results show that the use of functional module mapping enables us to correctly categorize organisms into main categories with specific metabolic characteristics. Traditional genome-based phylogenetic methods can reconstruct phylogenetic relationships, whereas our method can offer in-depth metabolic analysis for phylogenetic reconstruction, which can add insights into traditional phyletic reconstruction. The results also demonstrate that our phylogenetic trees are closer to the classic classifications in comparison to existing classification methods using metabolic pathway data.

## 1. Introduction

Phylogenetic reconstruction uses genetic information to reconstruct phylogenetic relationships among living organisms, which is a means to gain insight into the history of species and to retrospect the evolution of species. Some phylogenetic inference methods reconstructed phylogenetic trees based on the similarity of sequences of genes encoding 16S ribosomal RNAs and other marker genes [1]. In recent years, the quantity and quality of the metabolic pathway data have been greatly increased in biological databases like KEGG [2]. Comparative analysis of this vast quantity of metabolic pathway data provides a systematic way of exploring phylogenetic relationships between organisms, which has been demonstrated to be very effective for classifying organisms [1]. 

Much effort has been made to reconstruct phylogenetic trees in this way, which can be divided into two types. The first type aligns a single pair of metabolic pathways to compute the distance between organisms based on the similarity of enzymes [3], the similarity of reactions [4], and the topological similarity of pathways [5], and then creates phylogenetic trees using such distances. Since multiple metabolic pathways can provide more phylogenetic information than a single metabolic pathway, comparing multiple pathways among organisms can improve the accuracy of phylogenetic analysis. Following this motivation, the second type reconstructs phylogenetic trees by aligning multiple metabolic pathways among organisms. Mano et al. [6] adapted Meta Pathway Hunter (MPH) [7] to compare multiple metabolic pathways and analyze phylogenetic relationships for different organisms. Similarly, Ma et al. [1] employed IsoRankN [8], which is a global multiple-network alignment tool using spectral clustering methods, to investigate phylogenetic relationships from metabolic pathways. Subsequently, Clemente et al. extended the EC-based classification method to compute the structural similarity between pathways, and built phylogenetic trees by aligning all common metabolic pathways of different organisms [9]. 

In addition, some methods reconstructed phylogeny in other ways. For instance, Mazurie et al. computed phylogenetic distances by exploiting descriptors of metabolic reactions and obtained the phylogenetic trees that are similar to 16S rRNA-based trees [10]. Borenstein et al. provided a logical framework to infer the seed set of a given network and presented a seed method based on essential metabolites to reconstruct phylogenetic trees [11]. Chang et al. represented each organism as a vector of substrate-product relationships and reconstructed phylogenetic trees by comparing the vectors [12].

Although considerable progress has been achieved in reconstructing phylogeny, few efforts have focused on designing a unified phylogenetic framework that sufficiently reflects the metabolic features of organisms [6]. A functional module is a sub-network in metabolic pathway, which performs a certain metabolic function with specific topology [13]. Biologically, the metabolic features of organisms can be inferred from such functional module. Moreover, the topology and metabolic function of functional modules in common pathways are similar for the organisms from the same domain, whereas they may have certain differences for the organisms from different domains. And such differences may contain useful phylogenetic information. Therefore, comparative analysis of functional module mappings between multiple pathways can offer an effective way for building unified phylogenetic framework and revealing the metabolic features of organisms. 

In this paper, we propose a phylogenetic framework called MMAL that can characterize the metabolic features of organisms by aligning multiple metabolic pathways using functional module mapping. Our method transforms the alignment of multiple metabolic pathways into the construction of union graph of pathways using functional module mapping, which differs from the existing metabolic pathway alignment methods that directly compare the compounds and reactions in metabolic pathways. By clustering the nodes in the union graph, MMAL identifies the functional modules of pathways and build the mappings between these modules. Finally, MMAL computes the similarity between pathways by comparing the mapped functional modules in pathways, and infers phylogenetic relationships using the similarity. 

We demonstrated the effectiveness of MMAL by comparing resulting phylogenetic trees with the NCBI taxonomy. Note that the goal of our work is to explore the phyletic reconstruction from in-depth metabolic analysis, which cannot be afforded by the traditional classification scheme such as the NCBI taxonomy, and to provide insights into traditional phyletic reconstruction from the metabolism standpoint and offer a useful complement to the traditional phylogenetic methods. On the other hand, the NCBI taxonomy is a classic classification scheme that can be used to measure the quality of the resulting trees, and the aim of comparing resulting trees with the NCBI taxonomy is to evaluate the quality of the trees and represent the similarities and differences between resulting trees and classic classifications.

The experimental results show that the use of functional module mapping enables us to correctly categorize organisms into main categories with specific metabolic characteristics. Traditional genome-based phylogenetic methods can infer phylogenetic relationships, whereas our method can offer in-depth metabolic analysis for the phylogenetic reconstruction, which can add insights into traditional genome-based phyletic reconstruction. For example, by analyzing the resulting phylogenies, we have revealed that the metabolic structure of archaea is different from other species. The results also demonstrate that our classification results are consistent with the metabolic features of organisms and are closer to the classic classifications in comparison to existing classification methods using metabolic pathway data. 

## 2. Results

**Figure 1:**
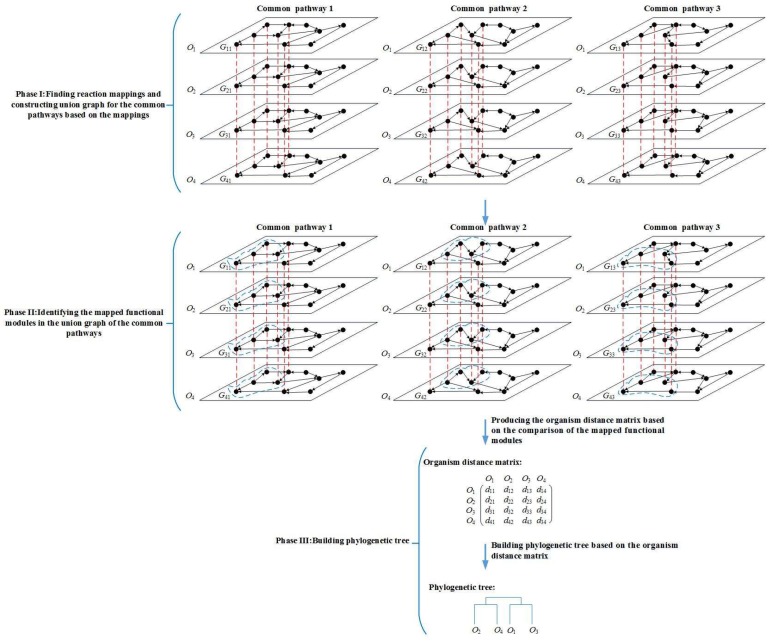
Overview of the MMAL method. MMAL builds the phylogenetic tree for 4 organisms by comparing the common pathways of these organisms in three phases. *G_ij_* is the common pathway *j* of organism *O_i_*, *i* = 1, 2, 3, 4, *j* = 1, 2, 3. The nodes in the pathways are reactions. The nodes connected with a red dashed line form a reaction mapping between pathways, and each reaction mapping constructs a composite node in the figure. The union graph is a graph that is constructed by the composite nodes and does not have edges. In the figure, each of common pathways 1, 2, and 3 has a union graph. In phase I, the union graph is constructed for common pathways 1, 2, and 3 respectively. In phase II, the mapped functional modules (the modules are composed of the nodes circled in blue dashed line in the figure) in the union graph of common pathways 1, 2, and 3 are identified. In phase III, MMAL obtains organism distance matrix from the comparison of the mapped functional modules, and builds the phylogenetic tree based on the matrix, wherein *d_i__j_* is the organism distance between organism *O_i_* and *O_j_*, *i* = 1, 2, 3, 4, *j* = 1, 2, 3, 4.

The overview of our method is summarized in Figure 1. In this work, the metabolic pathway data were retrieved from the KEGG database [2]. For given *k* organisms, each organism has *p* common metabolic pathways. We first perform MMAL to align these *k* × *p* metabolic pathways and produce a distance matrix of these *k* organisms. Then, based on the distance matrix, we build a phylogenetic tree for these organisms using the software tool PHYLIP [14], which is usually used to build the phylogenetic trees, and show the phylogenetic tree by the visualization tool TreeView [15]. The program kitsch in the PHYLIP package with the neighbor-joining algorithm was used to build a pathway-based phylogenetic tree from the distance matrix. To evaluate the quality of the produced trees, following [9], we use the well-known software package COUSINS [16] to compare the similarity between the produced tree and the NCBI taxonomy. This framework compares the trees based on cousin pairs: a sibling is a cousin of degree 0, a niece is a cousin of degree 0.5, a first cousin is a cousin of degree 1, and so on [16]. Then, two trees can be compared based on the set of pairs of each degree [16].

**Figure 2:**
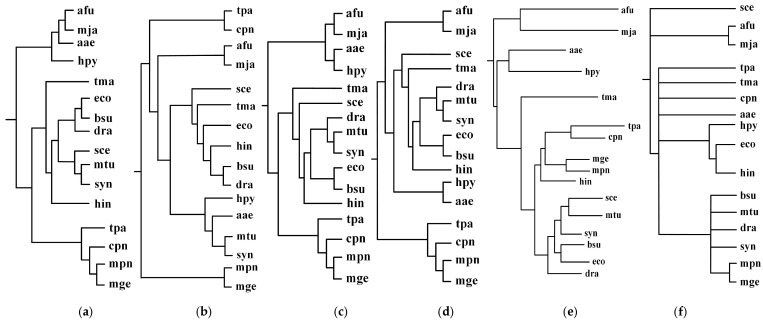
Incremental phylogenetic reconstruction for 16 organisms from their common metabolic pathways. (**a**) Our tree T_1_ based on aligning 5 common pathways for 16 organisms. (**b**) Our tree T_2_ based on aligning 10 common pathways for 16 organisms. (**c**) Our tree T_3_ based on aligning 15 common pathways for 16 organisms. (**d**) Our tree T_4_ based on aligning 20 common pathways for 16 organisms. (**e**) Heymans et al.’s tree for 16 organisms. (**f**) The NCBI taxonomy for 16 organisms.

**Figure 3:**
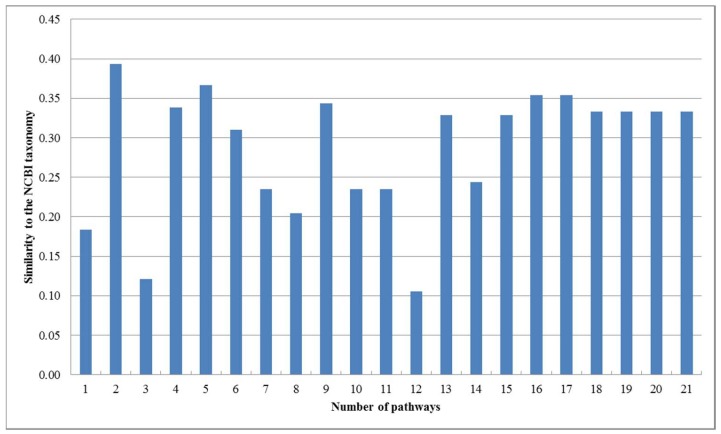
Similarities between our trees and the NCBI taxonomy for the 16 organisms.

First, we performed the incremental phylogenetic reconstruction of a set of organisms based on the comparison of all of their common metabolic pathways. Following [17], we have chosen 16 organisms: *Archaeoglobus fulgidus DSM 4304* (afu), *Methanocaldococcus jannaschii* (mja), *Aquifex aeolicus* (aae), *Helicobacter pylori 26695* (hpy), Thermotoga maritima (tma), *Treponema pallidum subsp. pallidum Nichols* (tpa), *Chlamydia pneumoniae CWL029* (cpn), *Mycoplasma genitalium G37* (mge), *Mycoplasma pneumoniae M129* (mpn), *Haemophilus influenzae Rd KW20* (hin), *Saccharomyces cerevisiae* (sce), *Synechocystis sp. PCC 6803* (syn), *Mycobacterium tuberculosis H37Rv* (mtu), *Bacillus subtilis* (bsu), *Escherichia coli K-12 MG1655* (eco), and *Deinococcus radiodurans* (dra). Heymans et al. [17] reconstructed a phylogenetic tree for these 16 organisms by comparing metabolic pathways, which is shown in Figure 2e. The similarity of Heymans et al.’s tree to the NCBI taxonomy is 0.26. We reconstructed the phylogenetic trees by aligning the common pathways of these organisms using the functional module mapping. Our produced trees T_1_, T_2_, T_3_, and T_4_ are shown in Figure 2, and the similarities of these produced trees to the NCBI taxonomy are shown in Figure 3.

As can be seen in Figure 2, our produced trees and Heymans et al.’s tree are similar to each other, and they are similar to the NCBI taxonomy, although there are some differences. The two mycoplasma mge and mpn are the two closest organisms and they are grouped together. The two archaea afu and mja are also clustered together. Comparing T_1_, T_2_, T_3_, and T_4_, we can find that their differences become smaller as the number of aligned pathways increases, which are consistent with the tendency of similarity values in Figure 3. Interestingly, the two archaea afu and mja always formed a single group in each case of our resulting trees, which suggests that the domain of archaea has particular characteristics in the metabolic pathways. This implies that the metabolic structure of archaea is different from that of other species. It is also interesting to observe from Figure 3 that, in 13 out of 21 cases, the similarities of our trees to the NCBI taxonomy are higher than 0.3, which are larger than the similarity between Heymans et al.’s tree and the NCBI taxonomy. This indicates that, although the overall classification results were similar, our method can produce better classifications for these 16 organisms in comparison to another phyletic method. 

On the other hand, as shown in Figure 3, the similarity of our produced tree to the NCBI taxonomy fluctuates when the number of pathways is less than 15. However, with the increasing number of pathways, the reconstructed trees had a tendency toward stable similarity to the NCBI taxonomy. This implies that the quality of the reconstructed tree is directly affected by the number of aligned pathways and remains stable when the number of pathways becomes large. 

**Figure 4:**
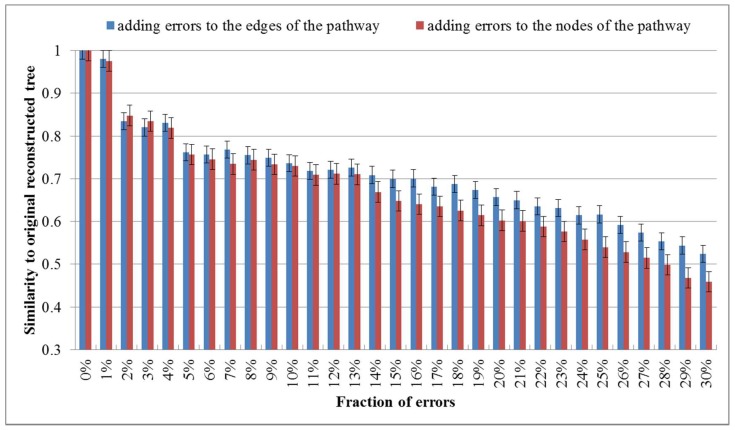
Average values of similarities between perturbed trees and the original one. The error rate increases from 0% to 30%.

Although the metabolic pathway data of the KEGG database are probably among the most reliable available datasets of this kind [18], some errors in the metabolic pathway data may occur and could affect the classification results. To investigate the robustness against such errors, we randomly added errors to the edges or nodes of each metabolic pathway by randomly modifying a fraction of nodes or edges (eliminating existing ones and/or making new ones). We then reconstructed the phylogenies for these 16 organisms by aligning the pathways with errors. The robustness check was performed by calculating the similarity of the trees based on the pathways with errors to the original tree. We performed the robustness check for each fraction of errors 10 times and showed the average values in Figure 4.

As shown in Figure 4, the similarity to the original tree decreases as the fraction of added errors increases and the similarity value becomes 0.8~0.85 when the error rate is 2%. This demonstrates that a moderate quantity of errors in the metabolic pathway data of the KEGG database affect the results in deed. Interestingly, with the continuously increasing error ratio, it can be seen that the similarity value falls even more clearly when the node errors goes up, which suggests that the impact of the node errors to the classification results is larger than that of the edge errors.

Since our phylogenetic trees are built based on the metabolic pathway data, in addition to comparing the resulting trees with the NCBI taxonomy, comparing our phylogenetic trees with the phylogenetic trees produced by other phylogenetic methods using metabolic pathway data can help to evaluate the effectiveness of our method from metabolisms. In the following, we analyze the resulting phylogenetic trees in detail and compare the quality of our produced trees with those constructed by competing methods [1,9,12,19] using metabolic pathway data.

### 2.1. Comparison with the Classification Based on Substrate-Product Relationships and the Classification Based on EC Hierarchy

EC hierarchy is the measure based on the enzyme hierarchy proposed by the Enzyme Commission of the International Union of Biochemistry and Molecular Biology, which can be used to assess the similarity of enzymes. In EC hierarchy, each enzyme is appointed a number, the EC number consisting of four digits, and the similarity of enzymes can be measured by comparing the EC number. Clemente et al. [9] used the EC hierarchy to define pathway similarity and study the relationship among eight photosynthetic bacteria and two photosynthetic eukaryotes by pseudo-alignment of over 60 metabolic pathways.

These photosynthetic bacteria are Anabaena sp. PCC7120 (ana), Gloeobacter violaceus (gvi), Prochlorococcus marinus SS120 (pma), Prochlorococcus marinus MED4 (pmm), Prochlorococcus marinus MIT 9313 (pmt), Synechocystis sp. PCC 6803 (syn), Synechococcus sp. WH8102 (syw), Thermosynechococcus elongatus (tel). The two photosynthetic eukaryotes are Arabidopsis thaliana (ath), Cyanidioschyzon merolae (cme). By aligning 57 common metabolic pathways for these 10 organisms, we reconstructed phylogenetic tree T_1_. For these 10 organisms, Chang et al. [12] represented each organism as a vector of substrate-product relationships and reconstructed phylogenetic tree T_2_ by comparing the vectors. Clemente et al. [9] reconstructed phylogenetic tree T_3_ by the EC hierarchy.

**Figure 5:**
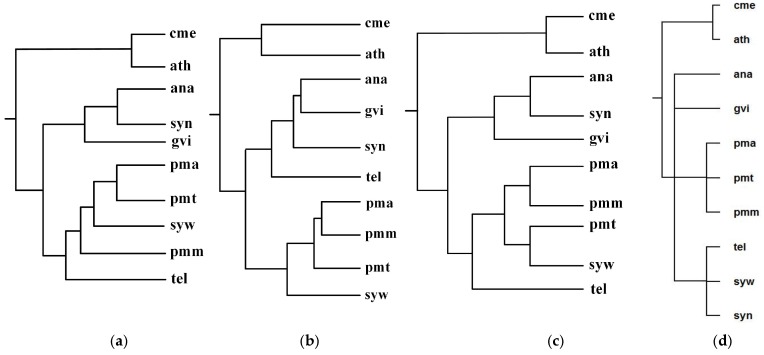
Phylogenetic trees for *Anabaena* (ana), *Gloeobacter violaceus* (gvi), *Prochlorococcus marinus* SS120 (pma), *Prochlorococcus marinus MED4* (pmm), *Prochlorococcus marinus MIT 9313* (pmt), *Synechocystis sp. PCC 6803* (syn), *Synechococcus sp. WH8102* (syw), and *Thermosynechococcus elongatus* (tel). (**a**) Our tree T_1_. (**b**) Chang et al.’s tree T_2_. (**c**) Clemente et al.’s tree T_3_. (**d**) The NCBI taxonomy T.

Figure 5 shows our produced tree T_1_, Chang et al.’s tree T_2_, Clemente et al.’s tree T_3_, and the NCBI taxonomy T for these 10 organisms. Table 1 shows the similarity measures of the reconstructed tree to the NCBI taxonomy T for these 10 organisms in Figure 5.

As can be seen from Figure 5, the classifications of T_1_, T_2_, and T_3_ are similar to each other. For instance, ana, gvi, and syn are grouped together to a branch, while pmm, pma, pmt, and syw are grouped together to another branch. We also can see that the classifications of gvi, syn and ana are similar in T_1_, T_2,_ and T_3_, whereas the branching of tel in T_2_ is different from that of T_1_ and T_3_. Chang et al.’s classification regards tel and gvi as metabolic out-groups in T_2_ because Chang et al. [12] indicated that gvi and tel were isolated from rocks and hot springs respectively, while the remaining six species were isolated from fresh or sea water. On the other hand, the common pathways of gvi, syn, and ana are similar in sequence and are different from the common pathways of tel, and therefore T_1_ and T_3_ separate tel from gvi, syn, and ana. In order to accurately separate the species with similar pathways, such as gvi, syn, and ana, a possible strategy is to combine genome features to the comparison of pathways, which is left as our future work. 

Since these eight photosynthetic bacteria share a large number of metabolic pathways with these two photosynthetic eukaryotes, it is extremely difficult to distinguish them by comparing their metabolic pathways alone [12]. However, as shown in Figure 5, by using the functional module mapping in the alignment of multiple pathways, our method constructed a phylogeny that distinguishes the photosynthetic eukaryotes from the photosynthetic bacteria, although it failed to separate the Chroococcales (syn, syw, tel) from the Prochlorales (pmm, pma, pmt), the Nostocales (ana), and the Gloeobacterales (gvi). This indicates that the use of functional module mapping depicts the metabolic characteristics of photosynthetic bacteria and photosynthetic eukaryotes and also contains a substantial quantity of phylogenetic information.

Meanwhile, upon detailed comparison of tree topologies, we can also observe that the classifications {cme, ath} and {pmt, pma} of T_1_, T_2_, and T_3_ are the same as T, while our produced tree T_1_ is more similar to T because the hierarchy of T_1_ is closer to T than T_2_ and T_3_. More precisely, as shown in Table 1, comparing T_1_, T_2_, and T_3_ to T, the similarity of T_1_ to T is 0.38, the similarity of T_2_ to T is 0.19, and the similarity of T_3_ to T is 0.16. This demonstrates that our method is capable of reconstructing the phylogenies that are closer to the NCBI taxonomy than another two classification methods for these 10 organisms.

### 2.2. Comparison with the Classification Based on Comparing Single Pair of Metabolic Pathways

MP-Align [19] is a typical classification method based on comparing single pair of metabolic pathways. In this section, we compare the phylogenetic tree produced by MMAL with the tree produced by MP-Align.

**Figure 6:**
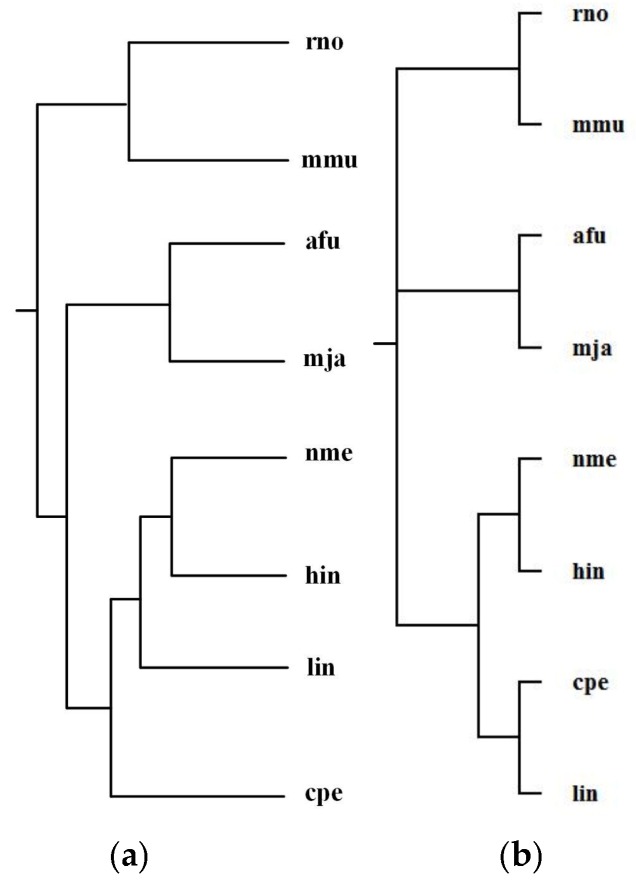
Phylogenetic trees for *Archaeoglobus fulgidus* (afu), *Clostridium perfringens* (cpe), *Haemophilus influenzae* (hin), *Listeria innocua* (lin), *Methanococcus jannaschii* (mja), *Mus musculus* (mmu), *Neisseria meningitidis* MC58 (nme), and *Rattus norvegicus* (rno). (**a**) Our tree T_1_ and the tree T_2_ constructed by MP-Align (T_1_ and T_2_ are the same). (**b**) The NCBI taxonomy T.

Alberich et al. [19] analyzed the phylogenies for the following 8 organisms: *Archaeoglobus fulgidus* (afu), *Clostridium perfringens* (cpe), *Haemophilus influenzae* (hin), *Listeria innocua* (lin), *Methanococcus jannaschii* (mja), *Mus musculus* (mmu), *Neisseria meningitidis MC58* (nme), and *Rattus norvegicus* (rno). The classification of these organisms is Bacteria (cpe, hin, lin, nme), Archaea (mja, afu), and Animals (mmu, rno). Alberich et al. performed the pairwise comparison of these 8 organisms for each common pathway and combined the computed scores to generate distance measures between these organisms and constructed phylogenetic tree T_2_ for the above 8 organisms. By aligning 47 common pathways for these 8 organisms, we reconstructed phylogenetic tree T_1_. Figure 6 shows our tree T_1_ and Alberich et al.’s tree T_2_ (T_1_ and T_2_ are the same) and the NCBI taxonomy T for these 8 organisms. The similarities of T_1_ and T_2_ to T are 0.31.

As can be observed in Figure 6, both MMAL and MP-Align do not recover exactly the phylogeny of the NCBI taxonomy, but they can correctly distinguish Bacteria, Archaea, and Animals and successfully classify the Bacteria into two distinct classifications {cpe, lin} and {hin, nme}, as in the NCBI taxonomy. Note that the two eukaryote rno and mmu are the two closest organisms, our method correctly grouped rno and mmu together and also placed the Bacteria cpe, hin, nme, and lin in a group separated from the other species. This implies that these parasitic bacteria show anomalous metabolism in comparison with other species, this is an interesting result which requires further investigation for its reason. 

### 2.3. Comparison with the Classification Based on Global Alignment of Multiple Metabolic Pathways

#### 2.3.1. Green Sulfur and Green Non-Sulfur Bacteria

**Figure 7:**
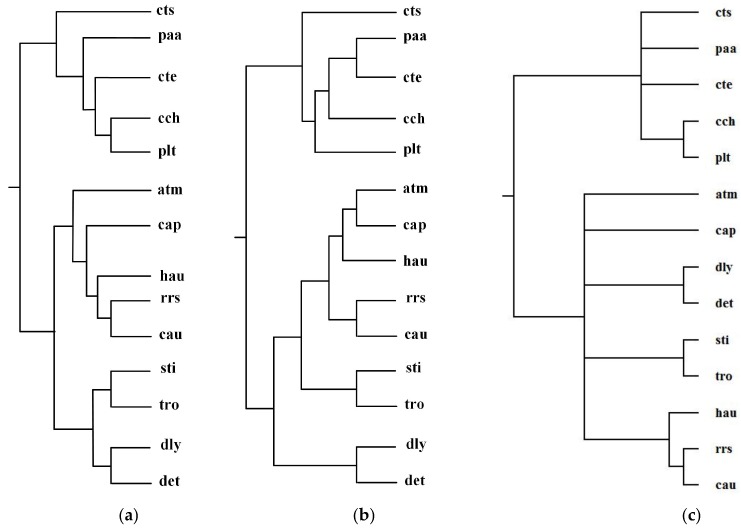
Phylogenetic trees for green sulfur and green non-sulfur bacteria. (**a**) Our tree T_1_. (**b**) Ma et al.’s tree T_2_. (**c**) The NCBI taxonomy T.

Green sulfur and green non-sulfur bacteria use two different sources of electrons in photosynthesis. Green sulfur bacteria use sulfide ion as the electron donor, whereas green non-sulfur bacteria do not [1]. Ma et al. selected 14 organisms from green sulfur and green non-sulfur bacteria and reconstructed phylogenetic tree T_2_ for these 14 organisms: *Anaerolinea thermophila*(atm), *Caldilinea aerophila* (cap), *Chloroflexus aurantiacus*(cau), *Dehalococcoides mccartyi 195* (det), *Dehalogenimonas lykanthroporepellens* (dly), *Herpetosiphon aurantiacus* (hau), *Roseiflexus sp. RS-1*(rrs), *Sphaerobacter thermophiles* (sti), *Thermomicrobium roseum*(tro), *Chlorobium chlorochromatii*(cch), *Chlorobaculum tepidum* (cte), *Chloroherpeton thalassium*(cts), *Prosthecochloris aestuarii* (paa), and *Pelodictyon luteolum* (plt). By aligning 52 common metabolic pathways for these 14 organisms, we reconstructed phylogenetic tree T_1_. Figure 7 displays our produced tree T_1_, and Ma et al.’s tree T_2_, and the NCBI taxonomy T for these 14 organisms. 

Table 2 shows the similarity measures of the reconstructed tree to the NCBI taxonomy for these 14 organisms in Figure 7.

In Figure 7, MMAL clearly separates these 14 bacteria into two broad metabolic categories: green sulfur bacteria and green non-sulfur bacteria. Green sulfur bacteria appear with the bacteria atm, cap, cau, det, dly, hau, rrs, sti, and tro. Green non-sulfur bacteria appear with the bacteria cch, cte, cts, paa, and plt. This classification result clearly characterizes the metabolic feature of green sulfur bacteria and green non-sulfur bacteria. Compared with T_2_, our produced tree T_1_ is more accurate. The reason is that as we can see from Table 2, the similarity of T_1_ to T is 0.20, whereas the similarity of T_2_ to T is 0.12. These results illustrate that our method is capable of classifying the organisms with specific metabolic characteristics, and it can obtain more accurate classification result than Ma et al.’s method for these 14 bacteria. 

#### 2.3.2. Prochlorococcus and Synechococcus

**Figure 8:**
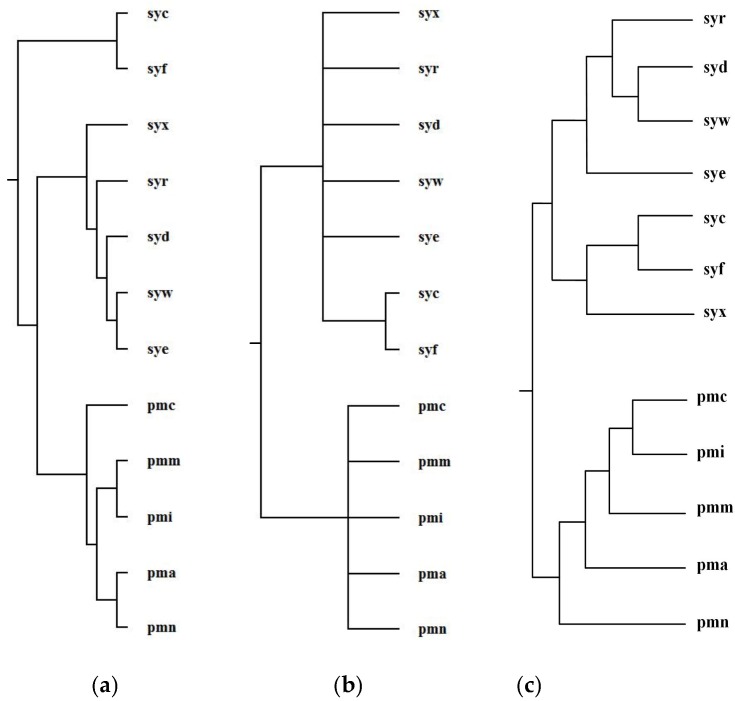
Phylogenetic trees for *Prochlorococcus* and *Synechococcus*. (**a**) Our tree T_1_. (**b**) Ma et al.’s tree T_2_. (**c**) The NCBI taxonomy T.

Ma et al. selected 12 organisms from *Prochlorococcus* and *Synechococcus* and reconstructed phylogenetic tree T_2_ for these 12 organisms [1]. For *Prochlorococcus* and *Synechococcus*, the similarity of their 16S rRNA sequences is greater than 0.96; however, they have different light-harvesting systems [1]. *Prochlorococcus* is composed of pmc (*Prochlorococcus marinus MIT 9515*), pmn (*Prochlorococcus marinus NATL2A*), pma (*Prochlorococcus marinus SS120*), pmi (*Prochlorococcus marinus MIT 9312*), and pmm (*Prochlorococcus marinus MED4*). *Synechococcus* is composed of syf (*Synech ococcuselongatus PCC 7942*), syc (*Synechococcuselongatus PCC 6301*), syx (*Synechococcus sp. WH7803*), syw (*Synechococcus sp. WH8102*), syd (*Synechococcus sp. CC9605*), syr (*Synechococcus sp. RCC307*), and sye (*Synechococcus sp. CC9902*). By aligning 64 common metabolic pathways for these 12 organisms, we reconstructed phylogenetic tree T_1_. Figure 8 shows our produced tree T_1_, and Ma et al.’s tree T_2_, and the NCBI taxonomy T for these 12 organisms. 

Table 3 shows the similarity measures of the reconstructed tree to the NCBI taxonomy T for these 12 organisms in Figure 8.

Concerning the organism pairs (pma, pmc), (syw, syx), and (pma, syx), the distinction between (pma, pmc) and (pma, syx) and the distinction between (syw, syx) and (pma, syx) are not obvious [1], which make it particularly hard to explicitly classify the species with high sequence similarity by the quantitative analysis [1]. As shown in Figure 8, MMAL successfully separated the organism pairs (pma, pmc) and (pma, syx) and separated the organism pairs (syw, syx) and (pma, syx) and correctly divided these 12 organisms into two broad metabolic categories *Prochlorococcus* and *Synechococcus*. This classification result clearly reflects specific metabolic characteristics among organisms and agrees well with the NCBI taxonomy. Moreover, the classification result of MMAL is more accurate than that of Ma et al.’s method. This is because, as we can observe from Table 3, the similarity of T_1_ to T is 0.14 whereas the similarity of T_2_ to T is 0.10. This illustrates that MMAL can reconstruct better phylogeny that is consistent with the metabolic features of organisms and close to the NCBI taxonomy for these 12 organisms.

## 3. Methods

For given *k* (*k* > 2) organisms, we try to determine the distance between the given organisms by aligning metabolic pathways, and build the phylogenetic tree for these *k* organisms based on the distance. The MMAL method consists of three main phases: Phase I—construct union graph of the common pathways of the given organisms (as detailed in Section 3.1). Phase II—identify the functional modules in the union graph of the common pathways (as detailed in Section 3.2). Phase III—build the phylogenetic tree through the mapped functional modules (as detailed in Section 3.3). The flowchart of our method is shown in Figure 1. In the following, we will describe the details of each phase in our method.

### 3.1. Phase I—Constructing Union Graph

In order to accurately and efficiently align the common pathways of given *k* organisms, we try to construct the union graph *G^U^* of these common pathways to accomplish the pathway alignments. Next, we describe the construction of the union graph *G^U^* in detail.

To start with, we introduce some definitions and notations. A directed graph *G_p_*=(*V*(*G_p_*),*E*(*G_p_*)) is used to denote metabolic pathway *P*, where *V*(*G_p_*) is the node set of *G_p_* and *E*(*G_p_*) is the directed edge set of *G_p_*, and each node in *V*(*G_p_*) represents a reaction *u_i_* in *P*, *i* = 1, 2, …,|*V*(*G_p_*)|. If an output compound of reaction *u_i_* is an input compound of reaction *u_j_*, there is a directed edge from *u_i_* to *u_j_*, *j* = 1, 2, …,|*V*(*G_p_*)|. If both *u_i_* and *u_j_* are reversible, there is also a directed edge from *u_j_* to *u_i_*. Similarly, we use directed graph *G_p_*′=(*V*(*G_p_*′),*E*(*G_p_*′)) to denote metabolic pathway *P*′.

In the following, we discuss the computation of similarity *S*(*u*,*v*). We adapt the similarity *S*(*u*,*v*), which was used to compute the similarity between nodes in metabolic pathways in [20,21], to compute the similarity between node *u* in *G_p_* and node *v* in *G_p_*′.

*S*(*u*,*v*) = *α* × *Esim*(*u_e_*,*v_e_*) + *β* × *Csim*(*u_ic_*,*v_ic_*) + *γ* × *Csim*(*u_oc_*,*v_oc_*) (1)

where *u_e_* is the enzyme catalyzing reaction *u*, *v_e_* is the enzyme catalyzing reaction *v*, *Esim*(*u_e_*,*v_e_*) is the similarity between enzyme *u_e_* and enzyme *v_e_*, *u_ic_*, and *v_ic_* are the input compounds of *u* and *v* respectively, and *u_oc_* and *v_oc_* are the output compounds of *u* and *v* respectively. 

We use the enzyme similarity score [17,20,21] and compound similarity scores [20,21] to calculate the enzyme similarity *Esim*(*u_e_*,*v_e_*) and the compound similarities *Csim*(*u_ic_*,*v_ic_*) and *Csim*(*u_oc_*,*v_oc_*) respectively. Specifically, the EC identifier of an enzyme consists of four digits. *Esim*(*u_e_*,*v_e_*) equals 1 if all the four digits of the EC identifier of two enzymes are the same, *Esim*(*u_e_*,*v_e_*) equals 0.75 if the first three digits are the same, *Esim*(*u_e_*,*v_e_*) equals 0.5 if the first two digits are the same, *Esim*(*u_e_*,*v_e_*) equals 0.25 if only the first digit is the same, and *Esim*(*u_e_*,*v_e_*) equals 0 if the first digit is different [17,20,21]. *Csim*(*u_ic_*,*v_ic_*) is the average compound similarity of *u_ic_* and *v_ic_*, and *Csim*(*u_oc_*,*v_oc_*) is the average compound similarity of *u_oc_* and *v_oc_*. For instance, if *C*_1_ and *C*_2_ are the input compounds of *u_ic_*, and *C*_3_ and *C*_4_ are the input compounds of *v_ic_*, then *Csim*(*u_ic_*,*v_ic_*) = {*sim*(*C*_1_, *C*_3_) + *sim*(*C*_1_, *C*_4_) + *sim*(*C*_2_, *C*_3_) + *sim*(*C*_2_, *C*_4_)}/4, where *sim*(*A*, *B*) is the compound similarity between compounds *A* and *B*. Similarly, we can compute *Csim*(*u_oc_*,*v_oc_*). The similarity scores of compounds are obtained from [22]. Parameters *α*, *β* and *γ* are used to control the balance between the weights of *Esim*(*u_e_*,*v_e_*), *Csim*(*u_ic_*,*v_ic_*) and *Csim*(*u_oc_*,*v_oc_*) with the constraint *α* + *β* + *γ* = 1. Here, we use *α* = 0.4, *β* = 0.3 and *γ* = 0.3. 

Next, we discuss how to create the union graph *G^u^* of two pathways *G_p_* and *G_p_*′. For edge-weighted bipartite graph *G_b_*, we regard *V*(*G_p_*) and *V*(*G_p_*′) as two partitions of *G_b_*, respectively. Each edge of *G_b_* corresponds to a one-to-one node mapping between *G_p_* and *G_p_*′. According to the similarity *S*(*u*, *v*) between nodes *u* and *v*, each edge connecting *u* and *v* in *G_b_* is assigned to a weight *S*(*u*, *v*).

Then, we employ the maximum-weight bipartite matching algorithm MWBM [23] to extract one-to-one node mappings between *G_p_* and *G_p_*′. Specifically, each time MWBM selects the edge of *G_b_* with the maximal weight and extends the resulting set with the corresponding node mapping for this edge. MWBM stops when there are no more edges to be selected. We use each one-to-one node mapping (*u*, *v*) in the resulting set of node mappings to produce a composite node *c_d* = {(*u*,*v*)| *u*∈*V*(*G_p_*), *v*∈*V*(*G_p_*′)}. Finally, we use these composite nodes to create the union graph *G^u^* of *G_p_* and *G_p_*′. 

**Figure 9:**
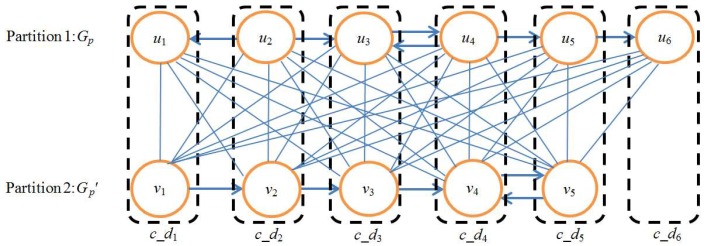
Example of union graph. The upper path is *G_p_* and the lower path is *G_p_*′. *u_i_*→*u_j_* denotes that the output compound of *u_i_* is the input compound of *u_j_*, *v_i_*→*v_j_* denotes that the output compound of *v_i_* is the input compound of *v_j_*, *i*, *j* = 1, 2, 3, 4, 5, 6. *V*(*G_p_*) and *V*(*G_p_*′) are regarded as two partitions of bipartite graph *G_b_*. Solid lines denote the edges of *G_b_*. Each edge of *G_b_* corresponds to a one-to-one node mapping between *G_p_* and *G_p_*′. The mappings in rectangles are the node mappings selected by the MWBM algorithm. Each selected node mapping constructs a composite node *c*_*d_i_*, *i* = 1, 2, 3, 4, 5, 6. A union graph of *G_p_* and *G_p_*′ is constructed by these six composite nodes.

Figure 9 shows an example of a union graph created for a pair of sample pathways. Note that there is no edge in the union graph.

After constructing the union graph *G^u^* of *G_p_* and *G_p_*′, we introduce how to compute the homological similarity between composite nodes in *G^u^* and build the homological similarity matrix of *G^u^*. Let the node set of *G^u^* be *V*(*G^u^*) = {*c*_*d*_1_, *c*_*d*_2_, …, *c*_*d_i_*, …, *c*_*d_n_*}, where *c*_*d_i_* is a composite node in *G^u^*, *i* = 1, 2, …, *n*, and *n* = max{|*V*(*G_p_*)|,|*V*(*G_p_*′)|}. We can compute the homological similarity *S_m_*(*c*_*d*_1_,*c*_*d*_2_) between composite nodes *c*_*d*_1_ and *c*_*d*_2_ by the following equation:

(2) $$S_{m}\left(c\_d_{1},c\_d_{2}\right)=\frac{1}{2}*\left(S\left(u_{1},v_{2}\right)+S\left(u_{2},v_{1}\right)\right)+\frac{1}{2}*\left(S\left(u_{1},v_{1}\right)+S\left(u_{2},v_{2}\right)\right)$$

where *u*_1_ and *v*_1_ are the nodes in mapping (*u*_1_, *v*_1_), *u*_2_ and *v*_2_ are the nodes in mapping (*u*_2_, *v*_2_), *u*_1_,*u*_2_ ∈ *V*(*G_p_*), *v*_1_,*v*_2_ ∈ *V*(*G_p_*′).

Subsequently, we construct an *n* × *n* homological similarity matrix *H^u^* by computing homological similarity between the composite nodes in *G^u^*, where element *H^u^*[*c*_*d_i_*,*c*_*d_j_*] ∈ [0,1] is the homological similarity between composite nodes *c*_*d_i_* and *c*_*d_j_*.

Now, we elaborate on how to construct the union graph *G^U^* and obtain the homological similarity matrix of *G^U^*. Let *G_i_*(*V_i_*, *E_i_*) be the common pathway of a given organism, *i* = 1, 2, …, *k*, and *G* = {*G*_1_(*V*_1_, *E*_1_), *G*_2_(*V*_2_, *E*_2_), …, *G_k_*(*V_k_*, *E_k_*)} be the set of the common pathways of given *k* organisms. 

We first select the metabolic pathway *G_max_* from *G*, where the number of nodes of *G_max_* is maximal in *G*. Second, we iteratively use *G_max_* and each metabolic pathway *G_i_* ∈ *G* to create a union graph *G_i_^u^* as well as its corresponding homological similarity matrix *H_i_^u^*, where the node set of *G_i_^u^* is *V*(*G_i_^u^*) = {*c*_*d*_1*i*_, *c*_*d*_2*i*_, …, *c*_*d_ni_*}, *i*= 1, …, *k*, and *n* = |*V*(*G_max_*)|. Every node in a composite node is mapped to a node of *G_max_*, and therefore any two nodes mapped to the same node of *G_max_* also construct a one-to-one node mapping. We thus build *k*-1 union graphs and the mappings between nodes in each pathway by using *G_max_*. 

To this end, we obtain *k* − 1 union graphs and *k* − 1 homological similarity matrices of these union graphs. Since each node of pathway *G_i_* ∈ *G* is mapped to a node of *G_max_* in each composite node in the union graphs, these *k* − 1 union graphs can be merged into a resulting union graph by merging the composite nodes which include the same node of *G_max_*. In this way, we finally merge these *k* − 1 union graphs into a resulting union graph *G^U^*, where the node set of *G^U^* is $V\left(G^{u}\right)=\left\{\cup_{i=1}^{k}c\_d_{1i},\cup_{i=1}^{k}c\_d_{2i},.....,\cup_{i=1}^{k}c\_d_{ni}\right\}$, and the homological similarity matrix of *G^U^* is $H^{U}=\sum_{i=1}^{k}H_{i}^{u}$.

Aligning metabolic pathways is to find the node mappings between pathways. Any two nodes (reactions) in a composite node in *G^U^* constitute a one-to-one node mapping between any two pathways in *G*. We can extract all one-to-one node mappings among the common pathways of these *k* organisms from *G^U^*. We thus transform the alignment of multiple pathways into constructing the resulting union graph *G^U^*.

### 3.2. Phase II—Identifying Functionally Conserved Modules

The goal of this phase is to obtain the functional modules and their mappings in the common pathways of given *k* organisms. A functional module is a sub-network in metabolic pathway, which performs a certain function with specific topology. We can cluster functionally similar composite nodes in the resulting union graph to obtain such a module. The induced sub-graph of the nodes of a resulting cluster in the underlying pathway is a functionally conserved module in the pathway.

In this work, we use affinity propagation (AP) algorithm [24] to cluster the functionally similar composite nodes in the resulting union graph based on the homological similarity matrix produced in Phase I. We use the homological similarity matrix *H^U^* as the input matrix to run the AP algorithm, and obtain a set of resulting clusters for the composite nodes of *G^U^*. Each of the resulting clusters is a set *U_M_* of composite nodes. For a metabolic pathway, each induced sub-graph of the nodes of a resulting cluster in the pathway is a functionally conserved module. For example, for the resulting union graph *G^U^* built in phase I, for simplicity, we assume that a resulting cluster *U_M_* = $U_{M}=\left\{\cup_{i=1}^{k}c\_d_{1i},\cup_{i=1}^{k}c\_d_{2i},\cup_{i=1}^{k}c\_d_{5i},\cup_{i=1}^{k}c\_d_{7i}\right\}$ is generated from $V\left(G^{u}\right)=\left\{\cup_{i=1}^{k}c\_d_{1i},\cup_{i=1}^{k}c\_d_{2i},.....,\cup_{i=1}^{k}c\_d_{ni}\right\}$ by using the AP algorithm based on the homological similarity matrix *H^U^*. For *G_i_*(*V_i_*,*E_i_*), the induced sub-graph of the nodes of *G_i_*(*V_i_*,*E_i_*) in *U_M_* is a functionally conserved module *M_i_* in *G_i_*(*V_i_*,*E_i_*), *i* = 1,2, …, *k*. We refer the readers to [24] for details on using the AP algorithm.

Meanwhile, since any two nodes of *M_i_* and *M_j_* in a composite node in *U_M_* construct a one-to-one node mapping between *M_i_* and *M_j_*, *M_i_*, and *M_j_* construct a one-to-one module mapping, where *i*, *j* = 1, 2, …, *k*, and *i* ≠ *j*. That is, if all nodes of two functional modules are included in the same resulting cluster, these two modules are mapped together. To this end, by clustering the composite nodes with similar functions in the resulting union graph *G^U^*, we identify the conserved modules and their mappings in the common pathways of *k* organisms.

### 3.3. Phase III—Building Phylogenetic Tree

Now, we describe how to create the distance matrix of *k* organisms by computing the similarity between the mapped functional modules in the pathways and build the phylogenetic tree based on the distance matrix.

For two metabolic pathways, the Largest Common Connected Sub-graph (LCCS) is the largest connected sub-graph of the first pathway that is isomorphic to a sub-graph of the second pathway [20]. The larger and denser connected sub-graphs are biologically more valuable. Larger numbers of nodes and edges of the LCCS in the mapped modules indicate that the LCCS in the mapped modules is larger and denser. Thus, we can use the number of nodes and edges of the LCCS in the largest mapped functional modules to measure the similarity between two pathways.

Next, we elaborate on how to measure the similarity between two pathways by comparing the similarity between the mapped functional modules in the pathways. For the pathways *G_i_*(*V_i_*,*E_i_*) and *G_j_*(*V_j_*,*E_j_*), let *Mmax_i_* and *Mmax_j_* be the mapped functional modules with the maximal number of nodes in *G_i_*(*V_i_*,*E_i_*) and *G_j_*(*V_j_*,*E_j_*) respectively. We define *M_lccs_i_* as the LCCS between *G_i_*(*V_i_*,*E_i_*) and *G_j_*(*V_j_*,*E_j_*) in *Mmax_i_*, where the node and edge sets of *M_lccs_i_* are *V_lccs_i_* and *E_lccs_i_* respectively. Similarly, we define *M_lccs_j_* as the LCCS between *G_i_*(*V_i_*,*E_i_*) and *G_j_*(*V_j_*,*E_j_*) in *Mmax_j_*, where the node and edge sets of *M_lccs_j_* are *V_lccs_j_* and *E_lccs_j_* respectively. The similarity score *SimScore*(*G_i_*,*G_j_*) between *G_i_*(*V_i_*,*E_i_*) and *G_j_*(*V_j_*,*E_j_*) is computed by equation (3).

(3) $$SimScore(G_{i},G_{j})=\frac{\min\left\{\left|E\_l_{ccs_{i}}\right|,\left|E\_l_{ccs_{j}}\right|\right\}}{\max\left\{\left|E_{i}\right|,\left|E_{j}\right|\right\}},i,j=1,2,.....,k.$$

In the following, we introduce how to compute the similarity between two organisms by the similarity between their common pathways. The given *k* organisms are denoted by *O*_1_, *O*_2_, …, and *O_k_*, and *p* common pathways of *O_i_* are represented by *G_i_*_1_, *G_i_*_2_, …, *G_ip_*, *i* = 1, 2, …, *k*. The similarity between *O_i_* and *O_j_* is computed by the following equation:

(4) $$SimScore(O_{i},O_{j})=\frac{\sum_{t=1}^{p}SimScore(G_{it},G_{jt})}{p},i,j=1,2,.....,k$$

Hence, by computing the similarity between any two organisms, we obtain an *k* × *k* similarity matrix *BSim* for given *k* organisms. *BSim* is a symmetric matrix. *BSim*[*i*,*j*] ∈ [0,1] is the similarity between *O_i_* and *O_j_*, *i* = 1, 2, …, *k,* and *j* = 1, 2, …, *k*. All elements in the diagonal of *BSim* are 1. 

After obtaining similarity matrix *BSim*, we can compute the distance matrix *D* for *k* organisms, where *D*[*i*,*j*] = 1 − *BSim*[*i*,*j*], *D*[*i*,*j*] is the distance between *O_i_* and *O_j_*, *i* = 1, 2, …, *k,* and *j* = 1, 2, …, *k*. Based on the distance matrix *D*, we can build a phylogenetic tree for these organisms using the software tool PHYLIP [14]. Finally, we can show this tree by the visualization tool TreeView [15].

## 4. Conclusions

Although a number of phylogenetic methods have been developed, few efforts were made to provide a unified phylogenetic framework that sufficiently reflects the metabolic features of organisms. We thus propose a three-phase phylogenetic method MMAL that can characterize the metabolic features of organisms by aligning multiple metabolic pathways using functional module mapping. 

MMAL distinguishes from other phylogenetic inference methods using metabolic pathway data in the following aspects. First, we transform the alignment of the metabolic pathways among multiple organisms into constructing the union graph of the pathways. Second, we identify the functional modules in the pathways and build the mappings between these modules simultaneously by clustering the nodes in the union graph. Finally, we compute the similarity between metabolic pathways by comparing the mapped functional modules in the pathways and construct the phylogenetic relationships among the organisms according to the similarity. 

We have shown the effectiveness of MMAL by comparing the resulting trees with the NCBI taxonomy. The experimental results demonstrate that the use of functional module mapping enables MMAL to categorize correctly organisms into main categories with specific metabolic characteristics and the classification results of MMAL are consistent with the metabolic features of organisms. Traditional phylogenetic methods can infer evolutionary relationships, whereas our method has the capacity to explore in-depth metabolic analysis for the phyletic reconstruction, which can add insights into traditional phyletic reconstruction. The results also show that MMAL is capable of reconstructing better phylogenies in comparison to existing classification method using metabolic pathway data. It is evident that investigating functional module mapping helps to construct better phylogenies. MMAL is thus a useful method for the study of phylogenetic analysis. 

The metabolic pathways are highly selected by diverse local environments, and therefore, some pathways of the organisms beyond a certain distance may be similar in sequence, which makes it difficult to distinguish the distant species with similar pathways by comparing metabolic pathways. Combining genome features, such as ribosomal RNAs and oligonucleotide compositions, to our phylogenetic framework offers a possible solution for this problem, and showing the history of the appearances of the functional modules can further improve the inference capability of MMAL, which would be of interest in our future study. Additionally, in this work, we have reconstructed phylogenies by aligning pathways based on one-to-one reaction mappings, and it would be interesting to reconstruct phylogenies by aligning pathways based on one-to-many reaction mappings.

Moreover, the study of phylogenetic relationships is not limited to metabolic networks but may also be applied to other biological networks such as protein interaction networks or transcriptional regulation networks. In the future, in order to understand the functional relations between different biological networks for different organisms, it is also interesting to quantitatively and qualitatively analyze the reconstruction of phylogeny from aligning different biological networks and explore the phenotypic differences between species from such alignments. 

## Figures and Tables

**Table 1:** Similarity of reconstructed tree to the NCBI taxonomy T for the organisms in Figure 5.

Reconstructed Tree	Similarity
Our tree T_1_	0.38
Chang et al.’s tree T_2_	0.19
Clemente et al.’s tree T_3_	0.16

**Table 2:** Similarity of reconstructed tree to the NCBI taxonomy T for the organisms in Figure 7.

Reconstructed Tree	Similarity
Our tree T_1_	0.20
Ma et al.’s tree T_2_	0.12

**Table 3:** Similarity of reconstructed tree to the NCBI taxonomy T for the organisms in Figure 8.

Reconstructed Tree	Similarity
Our tree T_1_	0.14
Ma et al.’s tree T_2_	0.10
